# S6. Proffered paper: Anti-tumour immunomodulatory activity of heat shock protein Hsp70 in therapy of malignant brain tumours: preclinical and clinical data

**DOI:** 10.1186/2051-1426-2-S2-I3

**Published:** 2014-03-12

**Authors:** M Shevtsov, A Kim, W Khachatryan, A Pozdnyakov, I Romanova, I Guzhova, B Margulis

**Affiliations:** 1 Institute of Cytology RAS, Saint-Petersburg, Russian Federation; 2 A.L. Polenov Russian Scientific Research Institute of Neurosurgery, Paediatric Neurosurgery, Saint-Petersburg, Russian Federation; 3 City Clinical Oncological Dispenser, Department of Radiology, Saint-Petersburg, Russian Federation; 4 I.M.Sechenov Institute of Evolutionary Physiology and Biochemistry RAS, Department of Morphology, Saint-Petersburg, Russian Federation

## Background

Immunotherapy provides a specific anti-tumour activity with minimal side effects and thus could be used in treatment of malignant brain tumours. Molecular chaperone Hsp70 is well known for its ability to stimulate innate and adaptive anti-cancer immune response. In in vivo studies we proved the efficacy of the intratumoral delivery of Hsp70 in the model of intracranial glioma C6 in animals. Based on our preclinical *in vivo* data we conducted for the first time pilot study for assessment of anti-tumour activity of Hsp70 in patients with brain tumours.

## Material and methods

12 patients with diagnosis of malignant brain tumour were enrolled in clinical trial. Following tumour resection protein was infused into tumor cavity. Specific immune response was evaluated in delayed hypersensitivity test (DTH). Peripheral blood was monitored for possible changes in lymphocyte subpopulations cytokine levels and cytolityc activity of NK-cells. Additionally cytokine production was analyzed in dynamics in liquor after injection of Hsp70. All patients were monitored for adverse effects.

## Results

Intratumoral injections of Hsp70 were well tolerated in patients. One patient had complete clinical response and one partial response documented by radiological findings. In three patients we observed positive DTH-test. In peripheral blood we observed a shift from cytokines of those provided by Th_2_-helpers towards cytokines from Th_1_-mediated response (i.e., INF-gamma, TNF-alpha). This data corresponded to changes in lymphocyte subpopulations. NK-cell activity was not altered.

## Conclusions

This pilot study demonstrated for the first time feasibility and safety of intratumoral delivery of recombinant Hsp70 in cancer patients. Based on our preclinical and clinical findings we proposed the novel hypothesis of immunomodulatory mechanism of Hsp70. Our results suggest that purified Hsp70 can induce specific effective anti-tumor immune response and warrants further investigation in randomised clinical trials.

